# Daytime sleepiness estimated using the Karolinska Sleepiness Scale during mandibular advancement device therapy for snoring and sleep apnea: a secondary analysis of a randomized controlled trial

**DOI:** 10.1007/s11325-025-03264-9

**Published:** 2025-02-18

**Authors:** Marie Marklund, Bo Carlberg, Lars Forsgren, Helene Rietz, Tommy Olsson, Karl A Franklin

**Affiliations:** 1 Department of Odontology, Faculty of Medicine, Umeå University, Umeå, SE-90187 Sweden; 2 Department of Public Health and Clinical Medicine, Medicine, Umeå University, Umeå, SE-90187 Sweden; 3 Department of Clinical Science, Umeå University, Neurosciences, Umeå, SE-90187 Sweden; 4 Department of Diagnostics and Intervention, Surgery, Umeå University, Umeå, SE-90187 Sweden

**Keywords:** Obstructive sleep apnea, Excessive daytime sleepiness, Symptoms, Mandibular Advancement Devices, Oral appliances

## Abstract

**Purpose:**

The effect of mandibular advancement device therapy on daytime sleepiness remains unclear. Here, we evaluate the effect of a mandibular advancement device on daytime sleepiness using the Karolinska Sleepiness Scale.

**Methods:**

We randomized 88 snoring patients with an apnea-hypopnea index < 30 and daytime sleepiness to a mandibular advancement device or a sham device for four months. The Karolinska Sleepiness Scale, which measures grades of sleepiness from 1 (very alert) to 9 (very sleepy), was used for seven consecutive days, four times each day. The results were analyzed with quantile regression at quartiles controlling for baseline, age, body mass index (kg/m^2^), sex, apnea-hypopnea index, and full-time work.

**Results:**

The Karolinska Sleepiness Scale score was lower with the mandibular advancement device than with the sham device at specific time intervals. The positive effect of mandibular advancement device therapy occurred at wake up and before lunch during the whole week and before lunch on weekdays at the middle quartile. The adjusted differences between the interventions favored mandibular advancement device therapy by almost one unit and normalized the Karolinska Sleepiness Scale scores at wake up and before lunch. In addition, there were positive effects of mandibular advancement device therapy before dinner at the highest quartile during the whole week, on weekdays, and on the weekend.

**Conclusion:**

Mandibular advancement devices used for snoring and sleep apnea reduce daytime sleepiness, particularly at wake up and before lunch, but provide some benefit before dinner.

**Supplementary Information:**

The online version contains supplementary material available at 10.1007/s11325-025-03264-9.

## Introduction

People who snore and suffer from obstructive sleep apnea often experience excessive daytime sleepiness [1, 2]. Sleepiness affects performance, mood, and quality of life and increases the risk for motor vehicle accidents 2–3 times [3].

Although a mandibular advancement device effectively reduces sleep apneas, its effect on daytime sleepiness remains unclear. We have earlier reported that a mandibular advancement device has no effect on daytime sleepiness [4], findings that are in line with most of the other identified randomized controlled trials we found [5–12] but not all [13–16]. All these studies used the Epworth Sleepiness Scale to evaluate daytime sleepiness. However, the Epworth Sleepiness Scale might not be a suitable measure for evaluating the effects of mandibular advancement device therapy, a treatment that is primarily recommended for patients with milder forms of the disease [17]. For example, this scale evaluates sleepiness retrospectively and summarizes answers to different types of questions addressing various degrees of daytime sleepiness [18–22]. In addition, repeatability is low when the Epworth Sleepiness Scale is used [23]. The Karolinska Sleepiness Scale, however, measures sleepiness with a very high individual stability at the time when the participant answers the scale [24–27]. In our study, we not only used the Epworth Sleepiness Scale but also analyzed the number of occasions that the patients reported sleepiness (Karolinska Sleepiness Scale score ≥ 7) at four different times of the day for seven consecutive days [4]. Only one other of these above trials used the Karolinska Sleepiness Scale in addition to the Epworth Sleepiness Scale [11]. Luz et al. [11] found that a mandibular advancement device had no effect on daytime sleepiness using the Epworth Sleepiness Scale or in evaluations for one day using the Karolinska Sleepiness Scale.

Here, we report a secondary analysis using Karolinska Sleepiness Scale on the data for our previously reported randomized controlled trial, where no estimations of daytime sleepiness at separate times of the day were made [4]. We aimed to evaluate the effect of mandibular advancement device therapy on sleepiness at different times of the day for seven consecutive days. We analyzed the results for the whole week and for weekdays and the weekend separately. Normative values of the Karolinska Sleepiness Scale score differ depending on the type of day in the week [27].

## Materials

### Study design

This randomized controlled trial uses parallel groups with two arms. Participants were randomized to receive a mandibular advancement device or a sham device and followed up after four months. The randomization was performed by a person outside the study staff with the help of a computer-generated table stratified for disease severity.

Patients responded to the Karolinska Sleepiness Scale immediately before they received either of the devices and then for seven consecutive days. Thereafter, they underwent polysomnographic sleep recordings. The measurements were repeated at the four-month follow-up.

### Participants

The sample consisted of 88 snoring patients (59 men) with an apnea-hypopnea index below 30 and daytime sleepiness who fulfilled the study protocol of the initially 96 randomized patients [4]. The inclusion criteria for daytime sleepiness were one or more of the following: Epworth Sleepiness Scale score ≥ 10; daytime sleepiness occurring “often” or “always”; unwillingly falling asleep during the daytime at least “sometimes” (on a scale “never”, “seldom”, “sometimes”, “often” and “always”); and “an irresistible tendency to fall asleep during the daytime one or more times per week” [28]. Exclusion criteria were tonsil hypertrophy criteria grade 3 or 4 on the Friedman scale, severe psychiatric diseases or dementia, untreated caries or periodontal disease, few teeth for anchoring a device, occupational drivers, participation in other studies, or patients with a bias with regard to the study. Three patients with shift work were excluded in the analysis.

### Outcomes

The measures of daytime sleepiness were made with the Karolinska Sleepiness Scale Version A [24, 26]. For seven consecutive days, the subjects rated their instant sleepiness in units from 1 (very alert) to 9 (very sleepy, fighting sleep) at four specific times during the day: in the morning when they woke up; at noon before lunch; in the afternoon before dinner; and at bedtime. The results are presented for the seven consecutive days–weekdays (Monday to Friday) and the weekend (Saturday and Sunday). In addition, the patients reported their sleep times, adherence, and side effects of the treatment.

### Statistical methods

Non-normally distributed data and ordinal data are described in the median and interquartile range (IQR). Normally distributed data are described as the mean and standard deviation (SD). Non-normally distributed data and ordinal data were analyzed with the Mann-Whitney U test for independent samples and Wilcoxon test for paired samples. Normally distributed data were analyzed with t-test for independent samples and paired samples t-test. The chi-squared test was used to analyze categorical data. Comparisons between the sham device and the active appliance on daytime sleepiness evaluated using the Karolinska Sleepiness Scale and the Epworth Sleepiness Scale were made with quantile regression analysis controlling for baseline, sex, age, body mass index (kg/m^2^), apnea-hypopnea index, and full-time work. The results were analyzed at the lowest quartile, the middle quartile, and the highest quartile during the whole week, on the weekdays, and on the weekend separately. The SPSS 28 statistical software package (IBM) was used in all calculations and a *p* < 0.05 was considered significant.

## Results

### Baseline characteristics

Baseline characteristics are described in Table 1. The subjects in the mandibular advancement device group were significantly younger than subjects in the sham device group (*p* = 0.019). There were no differences in baseline values of the Karolinska Sleepiness Scale scores or the Epworth Sleepiness Scale score between the mandibular advancement device group and the sham device group (Table 2).

**Table 1:** Demographic and polysomnographic characteristics at baseline

	MAD *n* = 44	Sham *n* = 44	*p*-value MAD vs. sham
Age (years)	49.9 (10.5)	54.8 (8.9)	0.019
BMI (kg/m^2^)	27.7 (3.5)	27.8 (3.6)	0.823
Apnea-hypopnea index	15 (7 to 22)	14 (7 to 21)	0.835
Women (%)	12 (27)	17 (39)	0.257
Epworth Sleepiness Scale	11 (8 to 15)	11 (8 to 13)	0.818

MAD = mandibular advancement device

Data reported in mean and standard deviation (SD), in median and interquartile range (IQR), and in percent

**Table 2:** Mandibular advancement device therapy compared with sham intervention on daytime sleepiness evaluated using the Karolinska Sleepiness Scale and the Epworth Sleepiness Scale

	MAD Baseline Median (IQR) *n* = 44	MAD 4 mo. Median (IQR) *n* = 44	Sham Baseline Median (IQR) *n* = 44	Sham 4 mo. Median (IQR) *n* = 44	Sham vs. MAD *p*-value	Lowest quartile† Sham-MAD (95%CI)	Middle quartile† Sham-MAD (95%CI)	Highest quartile† Sham-MAD (95%CI)
**Whole week**								
KSS at wake up	6.0 (5.0 to 7.0)	5.0*** (4.0 to 6.0)	7.0# (5.0 to 7.0)	6.0** (4.0 to 7.0)	0.200	0.8 (− 0.1 to 1.8)	**0.9** **(0.2 to 1.5)**	**0.8** **(0.1 to 1.4)**
KSS before lunch	5.0 (4.0 to 5.0)	3.5*** (3.0 to 4.8)	5.0# (4.0 to 5.0)	4.3** (3.0 to 5.0)	**0.027**	0.7 (− 0.0 to 1.3)	**0.6** **(0.0 to 1.1)**	0.5 (− 0.2 to 1.2)
KSS before dinner	5.0 (4.6 to 6.4)	4.5*** (3.0 to 5.0)	5.0# (5.0 to 7.0)	5.0** (3.0 to 6.0)	0.054	0.2 (− 0.5 to 1.0)	0.5 (− 0.1 to 1.1)	**0.8** **(0.1 to 1.6)**
KSS at bedtime	7.0 (6.0 to 7.0)	7.0 (5.3 to 7.0)	7.0# (7.0 to 8.0)	7.0 (6.6 to 7.0)	0.110	−0.1 (− 0.7 to 0.5)	0.3 (-0.3 to 0.8)	0.4 (− 0.1 to 0.9)
**Weekdays**								
KSS at wake up	7.0 (5.3 to 7.0)	5.0*** (4.3 to 7.0)	7.0# (5.0 to 7.0)	6.0** (4.0 to 7.0)	0.424	0.8 (− 0.2 to 1.9)	0.5 (− 0.5 to 1.4)	0.4 (− 0.4 to 1.1)
KSS before lunch	5.0 (4.0 to 5.0)	3.0*** (3.0 to 4.0)	5.0# (4.0 to 6.0)	4.0** (3.0 to 5.0)	**0.012**	**0.6** **(0.0 to 1.2)**	**0.8** **(0.3 to 1.3)**	**1.0** **(0.2 to 1.7)**
KSS before dinner	6.0 (5.0 to 7.0)	5.0*** (3.0 to 5.0)	5.0 # (5.0 to 7.0)	5.0* (3.0 to 6.0)	0.064	−0.1 (− 0.9 to 0.7)	0.4 (− 0.1 to 0.9)	**0.8** **(0.0 to 1.6)**
KSS at bedtime	7.0 (6.0 to 7.0)	6.5# (5.0 to 7.5)	7.0# (7.0 to 8.0)	7.0 (6.5 to 7.5)	0.078	0.7 (− 0.3 to 1.6)	0.4 (− 0.4 to 1.3)	0.5 (− 0.3 to 1.2)
**Weekend**								
KSS at wake up	6.0# (4.5 to 7.5)	4.5** (4.0 to 5.9)	5.5# (4.5 to 6.5)	5.0 (4.0 to 6.5)	0.376	0.4 (− 0.5 to 1.4)	0.7 (− 0.1 to 1.4)	0.6 (− 0.1 to 1.4)
KSS before lunch	4.5# (3.5 to 5.5)	4.0*# (2.5 to 5.0)	5.0# (4.0 to 5.5)	4.0 (3.0 to 5.4)	0.074	**0.7** **(0.2 to 1.3)**	0.5 (− 0.2 to 1.3)	0.6 (− 0.0 to 1.3)
KSS before dinner	5.0# (4.0 to 6.0)	4.5**# (3.0 to 5.0)	5.5# (4.0 to 6.0)	5.0 (3.6 to 6.0)	**0.024**	0.4 (− 0.4 to 1.2)	0.6 (− 0.2 to 1.3)	**1.1** **(0.4 to 1.7)**
KSS at bedtime	7.0# (6.5 to 7.5)	6.5*# (5.0 to 7.5)	7.5# (6.0 to 8.0)	7.0 (6.5 to 7.5)	0.078	0.4 (− 0.6 to 1.4)	0.1 (− 0.5 to 0.7)	0.5 (− 0.2 to 1.1)
ESS	11 (8 to 15)	6 (5 to 11)	11 (8 to 13)	10 (5 to 12)	0.129	0.8 (− 0.7 to 2.3)	0.6 (− 1.7 to 2.9)	**2.6** **(0.8 to 4.3)**

KSS = Karolinska Sleepiness Scale (from 1 to 9); ESS = Epworth Sleepiness Scale (from 0 to 24); MAD = mandibular advancement device; 95% CI = 95% confidence interval; IQR = interquartile range

† Quantile regression estimate (95%CI) adjusted for baseline, age, sex, body mass index (kg/m^2^), apnea-hypopnea index, and full-time work

# One subject missing

Within group significances: *p < 0.05; **p < 0.01; ***p < 0.001

### Daytime sleepiness during seven consecutive days in a week

**Fig. 1:**
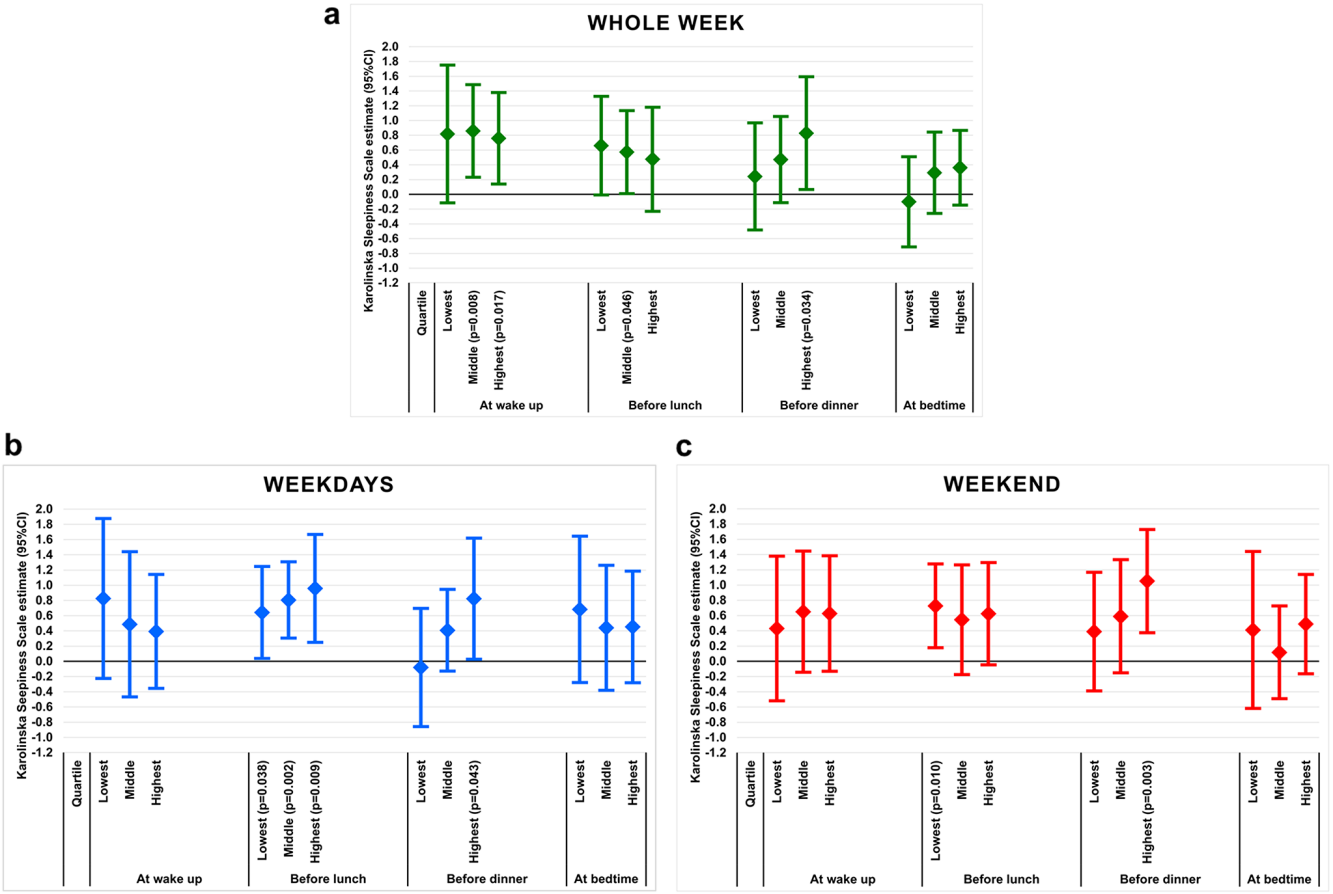
Average estimated differences (95% CI) in the Karolinska Sleepiness Scale score between the sham device group and the mandibular advancement device group after four months (**a)** for the whole week, (**b)** on weekdays, and (**c)** on the weekend

The Karolinska Sleepiness Scale score was significantly lower in patients using the mandibular advancement device than in those using the sham device at wake up (*p* = 0.008) and before lunch (*p* = 0.046) for the middle quartile controlled for baseline, sex, age, body mass index (kg/m^2^), apnea-hypopnea index, and full-time work (Table 2) (Fig. 1a). In addition, there were favorable effects of mandibular advancement device therapy at wake up (*p* = 0.017) and before dinner (*p* = 0.034) at the highest quartile of the outcome. The median Karolinska Sleepiness Scale score was reduced from 6.0 to 5.0 (*p* < 0.001) at wake up and from 5.0 to 3.5 (*p* < 0.001) before lunch in the mandibular advancement device group (Table 2). Boxplots of the Karolinska Sleepiness Scale scores at follow-up in the two intervention groups are presented in the Supplement (Fig. S1a).

### Daytime sleepiness on weekdays

The before lunch Karolinska Sleepiness Scale score was significantly lower in patients using the mandibular advancement device compared with those who used the sham device, with adjusted differences of 0.6 units (Quantile regression estimate) at the lowest quartile of the Karolinska Sleepiness Scale score (*p* = 0.038), 0.8 units at the middle quartile (*p* = 0.002), and 1.0 units at the highest quartile (*p* = 0.009) in favor of mandibular advancement device therapy (Table 2) (Fig. 1b). There was also a positive effect of the mandibular advancement device before dinner at the highest quartile (*p* = 0.043). The before lunch Karolinska Sleepiness Scale score decreased from 5.0 to 3.0 (*p* < 0.001) in the mandibular advancement device group (Table 2). Boxplots of the Karolinska Sleepiness Scale scores at follow-up in the two intervention groups are presented in the Supplement (Fig. S1b).

### Daytime sleepiness in the weekend

The Karolinska Sleepiness Scale score was significantly lower before lunch at the lowest quartile of the Karolinska Sleepiness Scale score (*p* = 0.010) and before dinner at the highest quartile (*p* = 0.003) than the sham device (Table 2) (Fig. 1c). The Karolinska Sleepiness Scale scores for the two groups are presented in Table 2 and Fig. S1c.

### Daytime sleepiness for all time points for seven consecutive days

The adjusted difference estimate (95%CI) between the interventions was 0.7 units (0.2 to 1.2) (*p* = 0.006) at the middle quartile of the Karolinska Sleepiness Scale score in favor of the mandibular advancement device therapy for all the summarized registrations at wake up, before lunch, before dinner, and at bedtime.

### Daytime sleepiness evaluated by the Epworth Sleepiness Scale

There was no difference in the numerical values of the Epworth Sleepiness Scale score between the two interventions at follow-up (Table 2). At the highest quartile, the Epworth Sleepiness Scale score was significantly lower in patients using the mandibular advancement device compared with those using the sham device, with an adjusted difference of 2.6 units (Quantile regression estimate) (*p* = 0.005) (Table 2).

### Subjective sleep, adherence, side effects, and the apnea-hypopnea index

Self-reported wake up time, sleep time, sleep latency, and naps did not differ between the interventions (Table 3). There were fewer awakenings during the nights with the mandibular advancement device than with the sham intervention (*p* = 0.003). Side effects were more common with the mandibular advancement device (*p* < 0.001). The median apnea-hypopnea index was 5 (2 to 11) (IQR) in the mandibular advancement device group and 17 (7 to 24) in the sham device group at follow-up (*p* < 0.001).

**Table 3:** Subjectively reported sleep, adherence, and side effects with the mandibular advancement device and the sham device

	MAD Baseline Median (IQR) *n* = 44	MAD Follow-up Median (IQR) *n* = 44	Sham Baseline Median (IQR) *n* = 44	Sham Follow-up Median (IQR) *n* = 44	*p*-value Between interventions
**Wake up time**					
Whole week	6:53 (6:21 to 7:14)#	6:44 (6:28 to 7:15)	7:00 (6:18 to 7:32)	6:32 (6:16 to 7:30)	0.556
Weekdays	6:20 (6:00 to 7:00)#	6:23 (6:00 to 6:53)	6:25 (5:51 to 7:00)	6:30 (6:00 to 7:16)	0.467
Weekend	8:00 (7:15 to 8:30)#	7:49 (7:11 to 8:45)	8:00 (7:05 to 8:45)#	7:56 (6:39 to 8:43)	0.570
**Sleep time**					
Whole week	7:15 (6:30 to 7:47)#	7:15 (6:41 to 7:56)	7:15 (6:30 to 8:00)#	7:20 (7:00 to 8:00)	0.280
Weekdays	7:00(6:30 to 7:45)#	7:00 (6:18 to 7:45)	7:00 (6:30 to 8:00)#	7:15 (7:00 to 8:00)	0.066
Weekend	8:35 (7:42 to 9:10)#	8:11 (7:15 to 8:45)	8:00 (6:45 to 8:52)#	7:57 (7:06 to 9:00)	0.943
**Sleep latency**	1 (0 to 2)##	0 (0 to 1)*###	1 (0 to 2)##	0 (0 to 1)####	0.148
**≥ 30 min (during week)**					
**Awakenings (per night)**	2 (1 to 3)#	1 (0 to 2)***	2 (1 to 4)#	2 (1 to 3)	**0.003**
**Naps (during week)**	2 (1 to 4)#	2 (0 to 3)*	1 (0 to 3)	1 (0 to 2)	0.085
**Use (nights)**		7 (6 to 7)		7 (5 to 7)	0.134
**Side effects (nights)**		1 (0 to 4)		0 (0 to 0)	**< 0.001**

MAD = mandibular advancement device; IQR = interquartile range

# One subject missing; ## Six subjects missing; ### Two subjects missing; #### Four subjects missing

Within group significances: *p < 0.05; **p < 0.01; ***p < 0.001

## Discussion

Mandibular advancement device therapy reduced daytime sleepiness at wake up and before lunch during the whole week and before lunch on weekdays compared with a sham device. This reduction was almost one unit of difference in the Karolinska Sleepiness Scale score. Throughout the use of the mandibular advancement device therapy, positive results were also observed before lunch and before dinner on the weekend. The positive effects of the mandibular advancement device on daytime sleepiness, as detected using the Karolinska Sleepiness Scale at different times of the day, revise the negative conclusions presented in our initial publication [4].

Luz et al. evaluated the effect of mandibular advancement device therapy using the Karolinska Sleepiness Scale but found no effect based on evaluations at five times on one day immediately after a polysomnographic sleep recording night [11]. The trial included 25 mild sleep apnea patients using mandibular advancement devices and 23 untreated controls in evaluations up to 12 months. We included 44 patients using the mandibular advancement device and 44 patients using a sham device and measured daytime sleepiness for seven consecutive days (i.e., one week) four times a day [4]. Our analyses revealed positive results for daytime sleepiness. Our polysomnographic sleep recordings were performed after the patients responded to the daily questionnaires to avoid a risk that the patients were sleepier than usual after the recording night. Therefore, we suggest that future trials use the Karolinska Sleepiness Scale repeatedly during the day and on several consecutive days, as this scale has the advantage to rate sleepiness at specific times during the day.

The Karolinska Sleepiness Scale allows for instant rating of all degrees of sleepiness, from its mildest forms to “very sleepy, fighting sleep”. Our subjects using the mandibular advancement device were less sleepy at wake up during the whole week and before lunch, particularly on weekdays, compared with those who used the sham device. In addition, there were positive effects before dinner among the patients who reported the highest degree of sleepiness during the interventions. The Epworth Sleepiness Scale, in contrast, measures the risk of dozing (i.e., a rather severe degree of sleepiness). This scale also depends on the subjects’ ability to remember their recent degree of sleepiness. For the present patients, daytime sleepiness was reduced only at the highest degree of sleepiness estimated by the Epworth Sleepiness Scale during the interventions. Therefore, our findings demonstrate that the Karolinska Sleepiness Scale compared with the Epworth Sleepiness Scale detects more positive effects of mandibular advancement device therapy. No studies were found in PubMed that address the effects on daytime sleepiness of continuous positive airway pressure or any other sleep apnea treatment evaluated using the Karolinska Sleepiness Scale. This scale could help fill in knowledge gaps about the effects of various sleep apnea treatments on daytime sleepiness and allow for the comparison of treatment modalities.

The numerical Karolinska Sleepiness Scale score at wake up was reduced from 6.0 at baseline to 5.0 at follow-up with the mandibular advancement device. The before lunch median baseline value of the Karolinska Sleepiness Scale was 5.0, both during the whole week and on weekdays. With the mandibular advancement device, this score was reduced to a median of 3.5 during the whole week and to 3.0 on weekdays. Normative values for the Karolinska Sleepiness Scale score in a European population are around 5 in the morning, 3–4 in the middle of the day, and 6 in the evening [25–27]. Untreated sleep apnea patients report around one unit higher on the Karolinska Sleepiness Scale score throughout the day [29]. Therefore, our findings demonstrate that mandibular advancement device therapy normalizes daytime sleepiness at wake up and before lunch.

Different effects of mandibular advancement device therapy were found on weekdays and on the weekend. On weekdays, the before lunch Karolinska Sleepiness Scale score was lower with the mandibular advancement device than with the sham device for all quartile levels (Fig. 1b). On the weekend, there were positive effects in patients with the lowest degree of sleepiness before lunch and with the highest degree of sleepiness before dinner (Fig. 1c). Normative values of the Karolinska Sleepiness Scale score are generally lower on days off compared with work days [27]. More varied types of activities during weekends compared with weekdays makes it recommendable to study treatment effects on daytime sleepiness for more than one weekend and consider the types of activities people engage.

One limitation in our study is that the results were found in a secondary analysis of the data. The subjects in the mandibular advancement device group were also younger than the subjects in the sham device group. However, it is unlikely that this difference would have influenced the results significantly. Older people have been found to be less sleepy than younger people, which primarily favor the sham device group [27]. Major strengths of this study include that the data come from a randomized controlled trial and that the scale–i.e., the Karolinska Sleepiness Scale–measures sleepiness when the participants answered the scale, which was done repeatedly on seven consecutive days. The use of this scale revealed novel findings of detailed treatment effects during mandibular advancement device therapy, including milder forms of daytime sleepiness.

## Conclusion

Mandibular advancement devices used for snoring and sleep apnea reduce daytime sleepiness, particularly at wake up and before lunch, but provide some benefits before dinner.

## Electronic supplementary material

Below is the link to the electronic supplementary material.

Supplementary Material 1

## Data Availability

The data that support the findings of this study are available from the corresponding author upon reasonable request.

## Declarations

### Clinical trial registration

TRIAL REGISTRATION clinicaltrials.gov Identifier: NCT00477009.
