# Supplementary material for: Daytime sleepiness estimated using the Karolinska Sleepiness Scale during mandibular advancement device therapy for snoring and sleep apnea: a secondary analysis of a randomized controlled trial
Source: Sleep Breath. 2025 Feb 18;29(1):107. doi: 10.1007/s11325-025-03264-9 (PMC11836080; doi:10.1007/s11325-025-03264-9)

SUPPLEMENT

**Daytime Sleepiness estimated using the Karolinska Sleepiness Scale during Mandibular Advancement Device Therapy for Snoring and Sleep Apnea: A Secondary Analysis of a Randomized Controlled Trial**

**Running title:** Sleepiness and mandibular advancement

Marie Marklund, Orthodontics, Department of Odontology, Faculty of Medicine, Umeå University, SE-90187 Umeå, Sweden. [marie.marklund@umu.se](mailto:marie.marklund@umu.se), marie.marklund@me.com

ORCID: 0000-0002-2927-000X

Bo Carlberg, Department of Public Health and Clinical Medicine, Medicine, Umeå University, SE-90187 Umeå, Sweden. bo.carlberg@umu.se

Lars Forsgren, Department of Clinical Science, Umeå University, Neurosciences, Umeå University, SE-90187 Umeå, Sweden. lars.forsgren@umu.se

Helene Rietz, Department of Public Health and Clinical Medicine, Medicine, Umeå University, SE-90187 Umeå, Sweden. [helene.rietz@umu.se](mailto:helene.rietz@umu.se)

Tommy Olsson, Department of Public Health and Clinical Medicine, Medicine, Umeå University, SE-90187 Umeå, Sweden. [tommy.g.olsson@umu.se](mailto:tommy.g.olsson@umu.se)

Karl A Franklin, Department of Diagnostics and Intervention, Surgery, Umeå University, SE-90187 Umeå, Sweden. karl.franklin@umu.se

**Supplementary Fig. S1 a, b and c**

Boxplots of the Karolinska Sleepiness Scale score in the sham device group and the mandibular advancement device group at wake up, before lunch, before dinner and at bedtime after 4 months *Abbreviation:* MAD, Mandibular advancement device

**S1a.** For the whole week


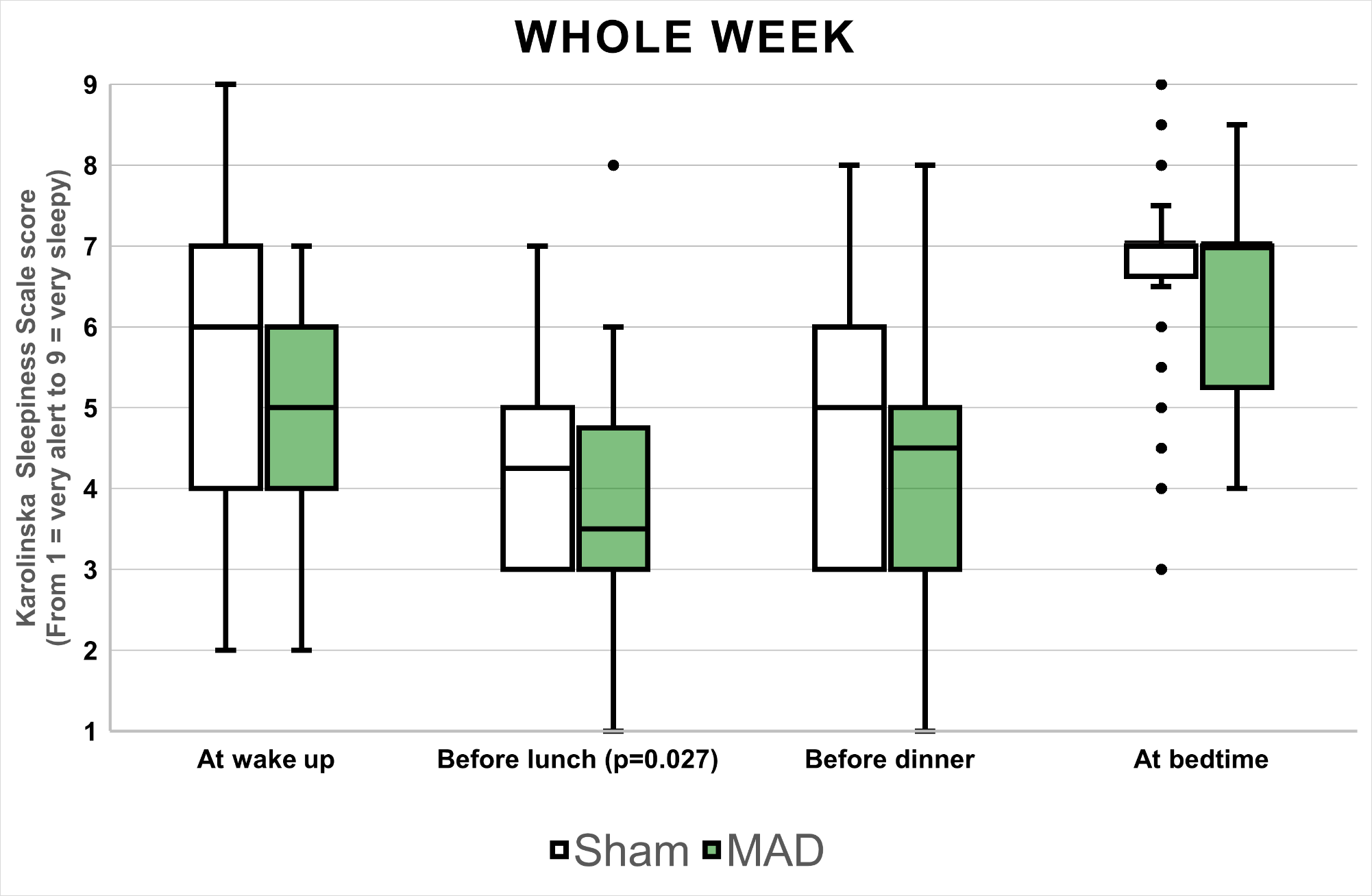


**S1b** On weekdays
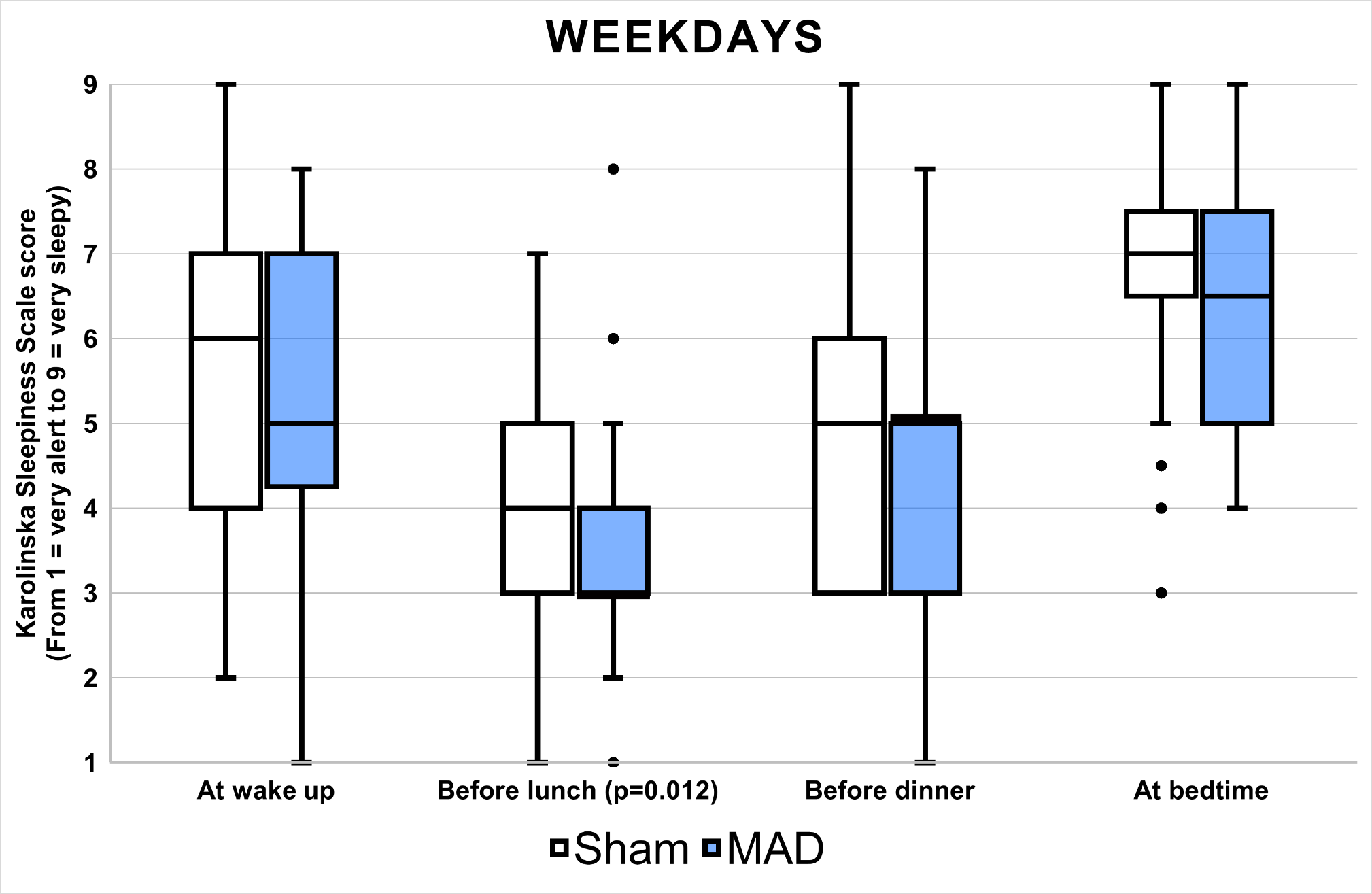


**S1c** On the weekend
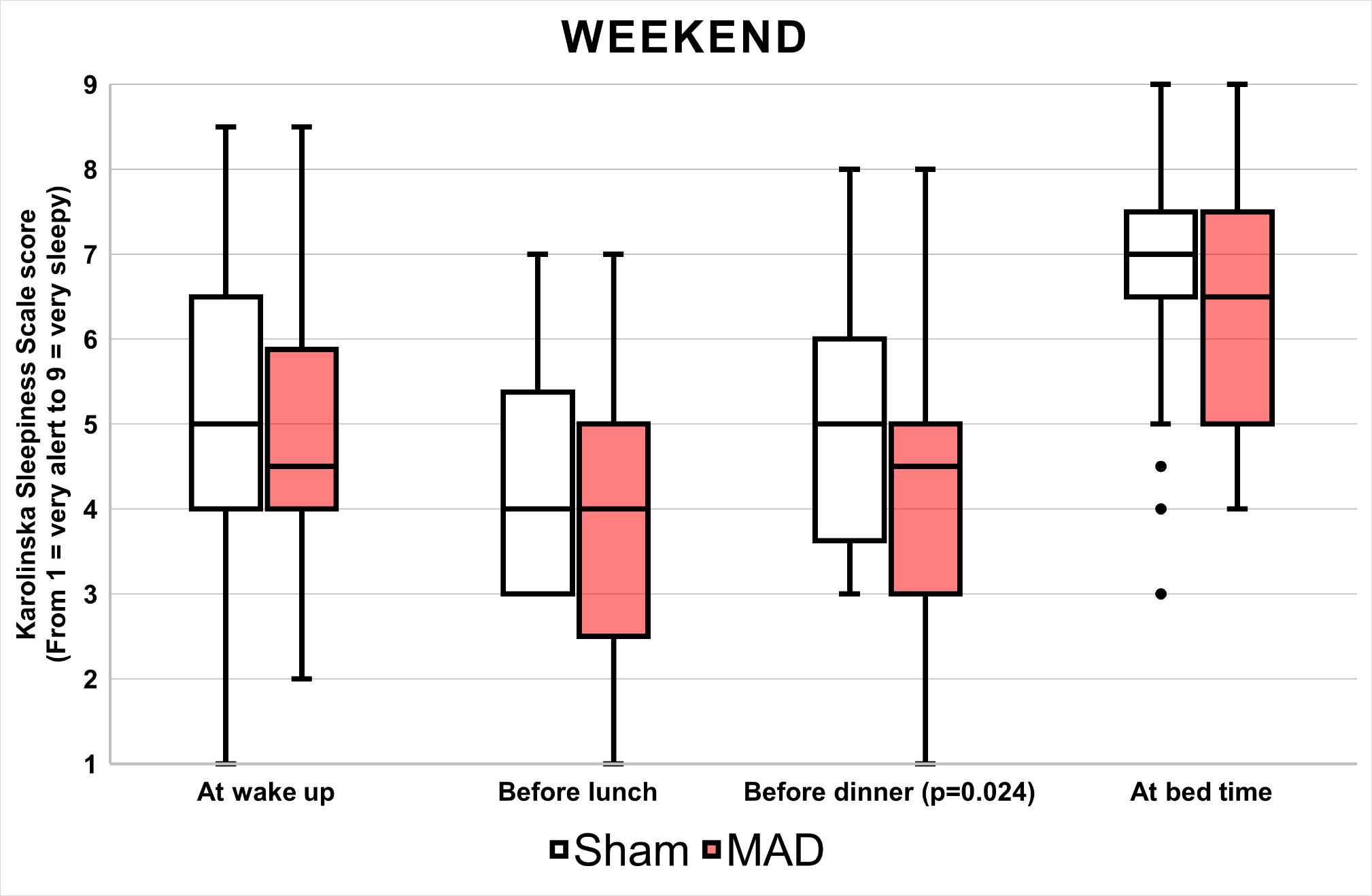

Supplement: Supplementary file 1 — Supplementary Material 1 [file 11325_2025_3264_MOESM1_ESM.docx]
